# Protocol for isolation and transplantation of rat pancreatic islets in the kidney capsule to treat diabetes

**DOI:** 10.1016/j.xpro.2024.103486

**Published:** 2024-12-13

**Authors:** Dayan Li, Weijing Kong, Yi Hong, Wei Zhang, Bingbing Sun, Xi Wang, Kai Wang

**Affiliations:** 1 Department of Physiology and Pathophysiology, School of Basic Medical Sciences, State Key Laboratory of Vascular Homeostasis and Remodeling, Clinical Stem Cell Research Center, Peking University Third Hospital, Peking University, Beijing 100191, China; 2 TianXinFu (Beijing) Medical Appliance Co., Ltd., Beijing 102200, China; 3 State Key Laboratory of Female Fertility Promotion, Department of Obstetrics and Gynecology, Peking University Third Hospital, Institute of Advanced Clinical Medicine, Peking University, Beijing 100191, China

**Keywords:** cell biology, cell isolation, health sciences, metabolism, model organisms, biotechnology and bioengineering

## Abstract

Islet transplantation offers promise for those with type 1 and late-stage type 2 diabetes; however, islet isolation from the pancreas is challenging due to the interference of exocrine tissue. We describe steps for constructing a diabetes model and precise islet isolation by a perfusion-based technique, followed by gradient centrifugation and cultivation in RPMI 1640 for recovery. Transplanted rat islets in diabetic nude mice demonstrated functional recovery, confirming the effectiveness of this approach.

## Highlights

• Introduction to the method of constructing a diabetes model in nude mice
• Instructions for pancreas dissection and collagenase P perfusion for islet harvest
• Protocol for high-yield islet purification by centrifugation and manual selection
• Guidance on functional islet transplant into the renal capsule to treat diabetes

## Abstract (editor highlights)

Publisher’s note: Undertaking any experimental protocol requires adherence to local institutional guidelines for laboratory safety and ethics.

## Graphical abstract

**Figure:**
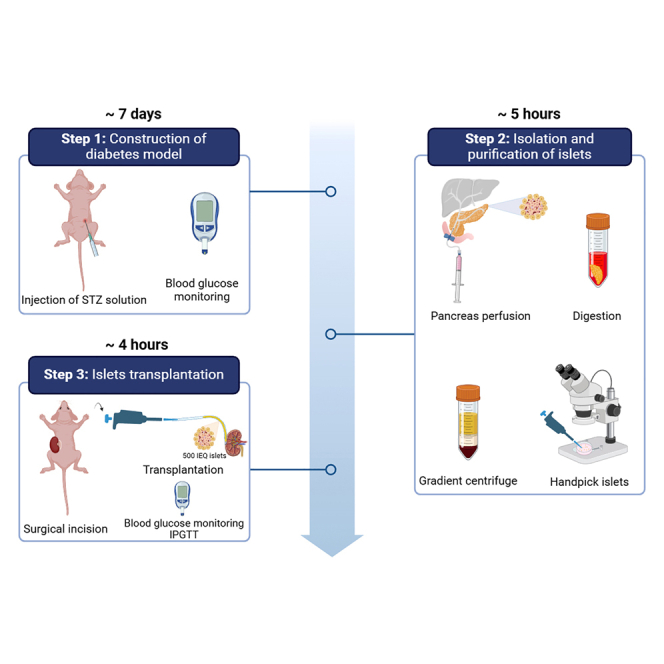

## Before you begin

In order to obtain a sufficient number of pancreatic islets for transplantation, we used a perfusion-based method to extract islets from rats, which increased the yield. To reduce the risk of rejection after transplantation, we induced diabetes in immunodeficient nude mice before transplanting rat islets into the renal subcapsular space of these diabetic mice. The process started with injecting streptozotocin (STZ) solution into the peritoneal cavity of nude mice to induce diabetes. After that, we perfused the rat pancreatic tissue with collagenase P solution and purified the islets using gradient centrifugation. Finally, we transplanted the isolated islets into the renal subcapsular space of diabetic nude mice, leading to the remission of diabetes in mice and confirming the functionality of the transplanted islets.^1^

### Institutional permissions

We utilized 10-week-old wild-type nude mice (weight >20 g) and SD rats (weight >250 g) for all experimental procedures. All animal protocols were approved by the authorities of Peking University and conducted according to local institutional guidelines and the principles outlined in the Animal Welfare Act. Before initiating this protocol, it is essential to ensure that all procedures involving animals and tissues have received approval from the relevant ethics review board.

## Key resources table

**Table:** 

REAGENT or RESOURCE	SOURCE	IDENTIFIER
**Chemicals, peptides, and recombinant proteins**		

Fetal bovine serum	Corning	Cat#35-080-cv
HBSS	Servicebio	Cat#G4206-500 mL
Streptozotocin (STZ)	YUA BIO	Cat#18883-66-4
D-glucose	Sigma	Cat#G8270-1 kg
Type-P collagenase	Sigma	Cat#11213873001
Citric acid monohydrate	Yeasen	Cat#60347ES25
Trisodium citrate dihydrate	Yeasen	Cat#60348ES25
RPMI 1640	Corning	Cat#10-040-cv
Penicillin-streptomycin solution	Procell	Cat#PB180120
Isoflurane	RWD	Cat#R-510-22-10
Histopaque 1077	Sigma	Cat#1077-1

**Experimental models: Organisms/strains**		

Male 10-week-old nude mice	Animal Department of Peking University Health Science Center	/
Male 8-week-old SD rats	Animal Department of Peking University Health Science Center	/

**Software and algorithms**		

Image-Pro Plus	Media Cybernetics	https://www.mediacy.com/imageproplus
GraphPad Prism 8	GraphPad Software	https://www.graphpad.com/scientific-software/prism/
Excel	Microsoft Office	https://www.office.com/
Adobe Illustrator 2021	Adobe	https://www.adobe.com/

**Other**		

Non-treated plate, 6 well	Nest	Cat#714001
Pipette	Eppendorf	Cat#H15024M
Centrifuge	Thermo Scientific	Cat#HEAEUS MULTIFUGE X3R
15 mL centrifuge tube	Labselect	Cat#CT-002-15
50 mL centrifuge tube	Jet	Cat#CFT011500
5 mL serological pipette	Jet	Cat#GSP010005
10 mL serological pipette	Jet	Cat#GSP010010
CO_2_ incubator	Thermo Scientific	Cat#3111-0019
Stereo microscope	OPTC	Cat#SZ680

## Materials and equipment

**Table:** Sodium Citrate Buffer, pH 4.5 (store at 4°C for 1 month)

Reagent	Final concentration	Amount
Trisodium citrate dihydrate	0.05 M	1.4705 g
Citric acid monohydrate	0.05 M	1.0505 g
Double distilled water	N/A	100 mL
Total	N/A	100 mL

**Table:** **STZ Solution** (prepare fresh every time)

Reagent	Final concentration	Amount
Sodium citrate buffer	N/A	50 mL
Streptozotocin (STZ)	10 mg/mL	500 mg
Total	N/A	50 mL

**Table:** **Collagenase stock solution** (store at −20°C up to 12 months)

Reagent	Final concentration	Amount
HBSS	N/A	450 mL
Type P collagenase	10 U/mL	2500 mg
Total	N/A	450 mL

***Note:*** The Collagenase P powder utilized in this protocol is supplied in a 2500 mg quantity. To minimize waste and ensure accuracy during preparation, the entire 2500 mg will be dissolved in 450 mL of HBSS solution. This results in a Collagenase P stock solution of 10 U/mL concentration. Subsequently, aliquots of 1–5 mL will be dispensed into 50 mL centrifuge tubes and stored at −80°C, which will be prepared into a working solution of 1 U/mL collagenase when needed.

**Table:** **Collagenase work solution** (prepare fresh every time)

Reagent	Final concentration	Amount
HBSS	N/A	45 mL
Collagenase stock solution	1 U/mL	5 mL
Total	N/A	50 mL

***Note:*** The Collagenase P work solution must be freshly prepared on ice. Each 1 mL of stock solution could be diluted to 10 mL of work solution using HBSS solution. The Collagenase P work solution must be sterile and free from endotoxins. The amount required for a single rat is 10 mL.

**Table:** **Neutralization buffer** (prepare fresh every time)

Reagent	Final concentration	Amount
HBSS	N/A	44.5 mL
Fetal Bovine Serum	10%	5 mL
Penicillin-streptomycin solution	1%	0.5 mL
Total	N/A	50 mL

**Table:** **Islets culture media** (store at 4°C up to 3 months, sterile)

Reagent	Final concentration	Amount
RPMI 1640	N/A	44.5 mL
Fetal Bovine Serum	10%	5 mL
Penicillin-streptomycin solution	1%	0.5 mL
Total	N/A	50 mL

## Step-by-step method details

### Induction of diabetes in nude mice

**Timing: 7 days**

In this section, diabetes is induced in nude mice. Nude mice are more susceptible to hypoglycemia and mortality when administered STZ solution intraperitoneally due to their weak immune systems. They are also more sensitive to STZ, especially at low ambient temperatures. Dose optimization studies in preliminary tests are essential to minimize mortality rates and improve the effectiveness of diabetes modeling.

1. Prior to administering the STZ solution, fast the experimental mice and replace their bedding entirely 24 h in advance.
2. Calculate the required volume of sodium citrate buffer and the mass of STZ base on the pre-weighed mice.

**CRITICAL:** STZ should be freshly prepared and stored in a light-proof brown tube on ice at all times. The STZ solution must be utilized within 10 min of preparation to prevent degradation.

***Note:*** The appropriate STZ dosage can elevate blood glucose levels in mice to between 16.7 mM and 27.8 mM within 7–10 days post-injection, with mice surviving for at least 3 months. Based on the relevant literature, the typical STZ injection dosage range for mice is generally between 130 and 150 mg/kg. Therefore, the dose optimization studies can be set up with three dose groups: 130 mg/kg, 140 mg/kg, and 150 mg/kg. The dose of 150 mg/kg will result in a 30% mortality rate in nude mice within 3 days post-injection, whereas the doses of 130 mg/kg and 140 mg/kg do not cause mortality, with the success rate for the dose of 140 mg/kg being higher than that of the 130 mg/kg dosage.

3. Administer STZ solution via intraperitoneal injection with a dosage of 140 mg/kg, based on the weight of each mouse.

**CRITICAL:** When injecting STZ solution intraperitoneally, be careful to avoid injecting into subcutaneous or intestinal tissues. To minimize the risk of puncturing internal organs, gently tilt the mouse's head 15° downward after securing it. Since the pancreas is located in the upper left abdominal region of the mouse, aim to insert the needle centrally into the left abdomen during injection. Try to deliver the STZ solution as close to the pancreas as possible. If initial attempts to induce diabetes are unsuccessful, consider prolonging fasting for an additional 2 h after administering the STZ solution.

4. Monitor the body weight and blood glucose levels of each mouse once per day during the first week after the STZ injection. Categorize the mice with blood glucose levels consistently above 16.7 mM for 2 consecutive days as successfully induced diabetic models.

### Isolation and purification of islets

**Timing: 5 h**

In this section, the pancreas is perfused with Collagenase P solution, isolated and extracted from the rats. Islets of the pancreas are divided through enzyme digestion, centrifugation and hand-pick. Finally, divided islets are incubated for further experiments.

**Figure 1:**
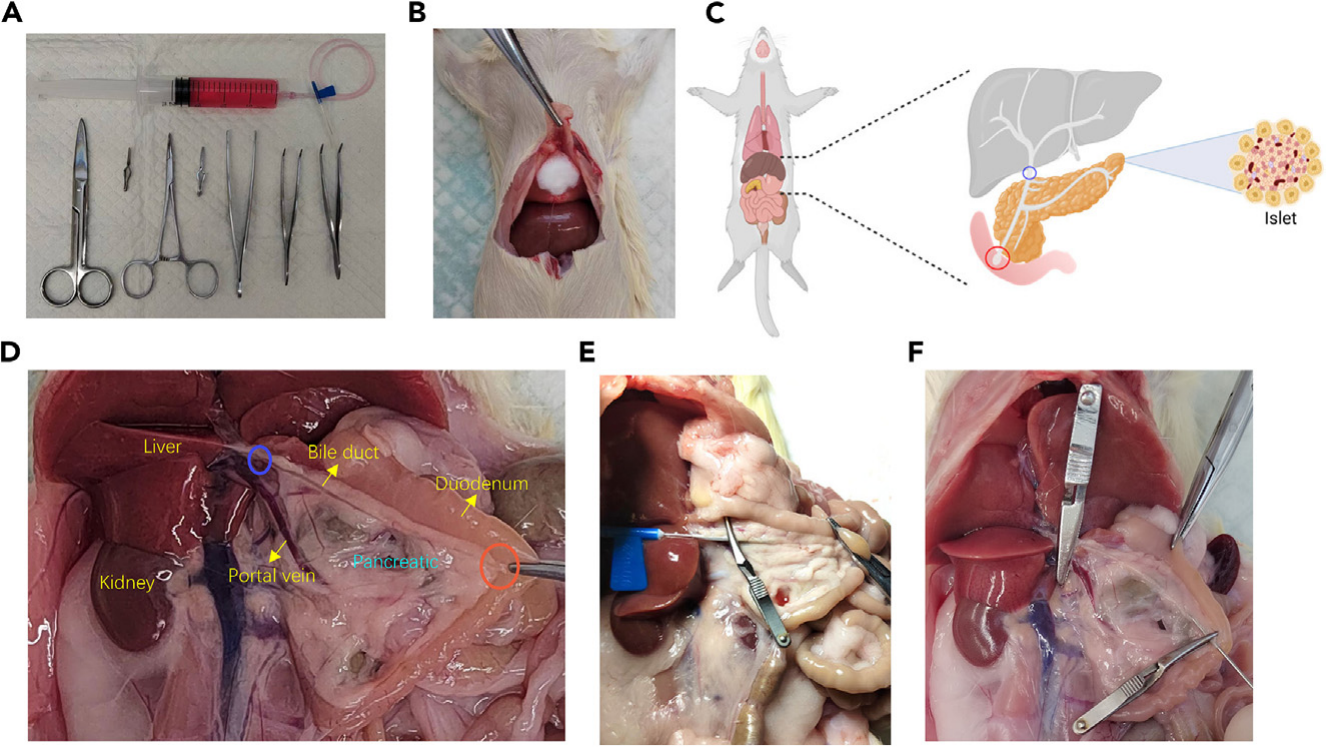
Pancreas perfusion (A) Primary surgical instruments used during the procedure. (B) Incision of the thoracic aorta from the rat diaphragm opening to initiate blood release (immediate occlusion of blood outflow using a defatted cotton ball post-incision). (C) Schematic diagram of anatomical location of rat pancreas region. (D) Anatomical position of the pancreatic region in the rat. (E) Insert an intravenous injection needle into the common bile duct near the liver to perfuse the pancreas. (F) Insert an intravenous injection needle into the common bile duct near the duodenum to perfuse the pancreas.

5. Sterilize surgical instruments such as surgical scissors, toothed forceps (length >10 cm), hemostatic forceps, ophthalmic forceps, and needle holders. Prepare 50 mL tubes, 75% alcohol, a thermos box with ice, and medical swabs (Figure 1A).
6. Set the water bath temperature to 37°C. Replace the centrifuge rotor capable of holding 50 mL tubes and cool it to 4°C.
7. Prepare Neutralization buffer by adding 10% fetal bovine serum (FBS) and 1% Penicillin-streptomycin solution to the HBSS solution.
  a. Prepare 2 bottles of 50 mL Neutralization buffer.
  b. Dilute the Collagenase P stock solution (10 U/mL) to a working concentration of 1 U/mL using HBSS solution.
  c. Keep the Neutralization buffer and Collagenase P work solution on ice.
8. Prepare RPMI 1640 medium containing 10% FBS and 1% Penicillin-streptomycin solution. Store the prepared medium in the refrigerator (4°C).
9. After placing the rat into the induction chamber of an anesthesia machine, confirm its unresponsiveness to toe pinching using toothed forceps. Subsequently, proceed with cervical dislocation.
10. Sever the thoracic aorta and inferior vena cava through surgery to prevent hemorrhage when extracting the pancreas.
  a. Secure the rats on a dissection board with limbs facing upward and tails toward the operator.
  b. Sterilize the abdominal skin and the area near the xiphisternum with 75% alcohol to prevent hair from adhering to surgical instruments during dissection.
  c. Use a hooked forceps to secure the xiphisternum, make an incision in the skin above it, and create a 1 cm longitudinal cut across the diaphragm without damaging surrounding tissues.
  d. Insert scissors into the thoracic cavity through the incision, sever the thoracic aorta and inferior vena cava, and promptly insert absorbent paper to manage bleeding.
  e. Replace the absorbent paper as needed when saturated with blood (Figure 1B).
11. Identify, isolate and clamp the Oddi’s sphincter to prepare for Collagenase P perfusion.
  a. Use hooked forceps to secure the skin around the urethra of the rat.
  b. Use surgical scissors to open the abdominal cavity from this point, exposing the intestines, liver, stomach, and spleen.
  c. Gently manipulate the liver towards the chest using smooth forceps to reveal the common bile duct and portal vein (Figure 1C).
  d. Identify Oddi’s sphincter along the common bile duct, a bulging structure near its junction with the duodenum.
  e. Use hemostatic forceps to clamp Oddi’s sphincter and the duodenum to prevent leakage of Collagenase P work solution during perfusion (Figure 1D).
12. Insert the needle with perfusion solution into the common bile duct.
  a. Draw 10 mL of Collagenase P work solution into a 20 mL syringe connected to a 23 G intravenous needle.
  b. Remove air from the syringe and tubing.
  c. Rotate the rat fixation plate 180° so the rat’s head faces the operator.
  d. Insert the intravenous needle near the liver into the section of the common bile duct and slowly inject 1 mL of collagenase work solution.
  e. Confirm correct needle placement by observing fluid flow into the bile duct.
  f. Clamp the needle and bile duct with hemostatic forceps to prevent solution leakage and backflow into the liver (Figure 1E).

***Optional:*** An alternative perfusion method involves clamping the bile duct near the liver, inserting a venous catheter into the duodenum, and performing perfusion with a volume of 10 mL. (Figure 1F).

**CRITICAL:** The original needle of a 20 mL syringe is usually 12 G, which is too thick for the common bile duct. As the common bile duct ruptures easily under the original needle of the syringe, we use a 23 G intravenous needle with an epitaxial tube for insertion into the bile duct, and the needle is connected to a 20 mL syringe (the original needle removed). The syringe and the intravenous needle can be pre-cooled on ice in advance to prevent the inactivation of collagenase activity.

13. Perfuse the pancreas with Collagenase P work solution.
  a. Perfuse the remaining Collagenase P work solution into the pancreas at a consistent rate until the pancreas visibly swells and becomes translucent.
  b. Gently manipulate the duodenum to ensure thorough perfusion of the pancreas tail near the spleen.
  c. If complete perfusion is not achieved, aspirate an additional 2 mL of collagenase P work solution and continue perfusion until the entire pancreas tail is adequately filled.

**CRITICAL:** Carefully observe the pancreas bulging without withdrawing the needle from the common bile duct initially, ensuring the needle does not inadvertently puncture the organ during manipulation.

**Figure 2:**
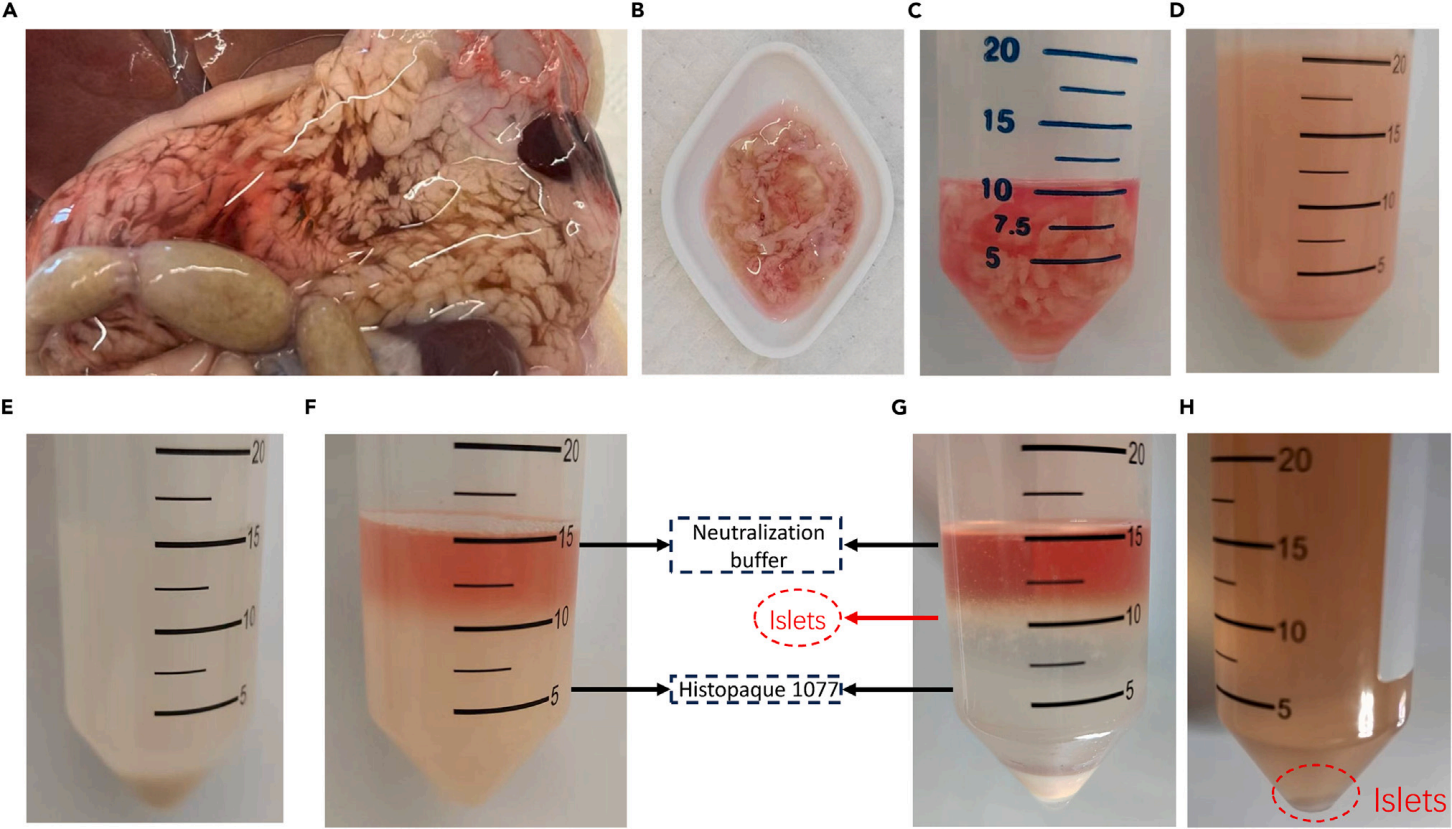
Pancreas perfusion and islets collection (A) The fully perfused pancreas. (B) The pancreas was dissected after perfusion. (C) Pancreas stored in a 50-mL centrifuge tube on ice before digestion. (D) Pancreatic tissue after digestion and centrifugation. (E) Pancreatic tissue after removal of adipose tissue and undigested tissue. (F) Pancreatic tissue suspension before purification using 1077. (G) Pancreatic tissue suspension after purification using 1077. (H) Washing of islets to remove residual Histopaque 1077 and subsequent centrifugation to obtain purified islets.

14. Strip and extract the pancreas from the abdominal cavity.
  a. Gradually remove the hemostatic forceps securing the needle, withdraw the needle from the common bile duct, and observe the inflated pancreas connected to the large intestine, small intestine, stomach, and spleen (Figure 2A).
  b. Begin by detaching the pancreas from the colon. Secure the rat’s colon with smooth forceps in the left hand and gently dissect the ligaments between the pancreas and intestines using ophthalmic forceps in the right hand.
  c. Proceed to strip the pancreas upward along the large intestine, small intestine, stomach, and spleen, then sever the bile ducts and hepatic portal vein (Figure 2B).
  d. Place the stripped pancreas into a 50 mL tube (Figure 2C).

**CRITICAL:** During pancreas extraction, minimize tissue damage and avoid squeezing to preserve the perfusion solution. The pancreas volume post-perfusion should ideally range from 5–10 mL, with a larger volume indicating more effective perfusion. To optimize islet yield, prevent bleeding, and separate the pancreas from adipose tissue meticulously during extraction. Pre-wet the interior walls of the 50 mL tube with pre-cooled collagenase work solution to prevent pancreas adherence.

15. After perfusing the pancreas of each rat, place the tube containing the pancreas on ice. Repeat the procedure for subsequent rats, and place their pancreas in the same 50 mL tube for digestion and purification.

**CRITICAL:** Do not place more than two pancreases in a single tube to maintain optimal purification efficiency. To prevent excessive collagenase digestion, ensure no more than 1 h elapse from the completion of perfusion to the start of digestion. Throughout the process, keep all samples on ice.

16. Add 3–5 mL of Collagenase P work solution to fully submerge the pancreas in the 50 mL tube. Place the tube in a 37°C water bath and digest for 30 min.
17. Immediately after digestion, transfer the tube to ice and terminate the digestion by adding 25 mL of cold Neutralization buffer (12 mL per rat). Shake the tube manually for 15 s to facilitate tissue breakdown.
18. Wash the pellet with Neutralization buffer.
  a. Centrifuge at 180 *g* at 4°C for 1 min.
  b. Carefully decant the supernatant.
  c. Add another 25 mL of Neutralization buffer and gently vortex until cells are uniformly suspended.
  d. Repeat the Neutralization buffer addition, shaking, centrifugation, and resuspension twice to ensure thorough washing of the pellet (Figure 2D).
19. Resuspend the pellet in 25 mL of Neutralization buffer and filter through a 425 μm diameter metal strainer to remove fat and undigested tissue.

***Note:*** Metal filters must undergo steam sterilization at high pressure and temperature followed by drying in an oven at 65°C before use. Pre-wetting the filters with 3 mL of Neutralization buffer before filtering the tissue suspension helps minimize the adherence of islets to the filter. After filtration, an additional 10 mL of Neutralization buffer can be used to rinse the filter and centrifuge tube to enhance islet yield.

20. Centrifuge at 180 *g* for 2 min at 4°C (Figure 2E). Carefully decant the supernatant and invert the tube on a paper towel for 5 min to drain excess liquid.
21. Resuspend the pellet with 1077 solution before adding the Neutralization buffer.
  a. Resuspend the pellet in 5 mL of 1077 solution.
  b. Vortex until the suspension is homogeneous.
  c. Rinse the tube wall with another 5 mL of 1077 solution to prevent islet adherence.
  d. Tilt the centrifuge tube at a 30-degree angle and gently pour 5–10 mL of Neutralization buffer along the tube wall.
  e. Maintain a clear demarcation line between the 1077 and Neutralization buffer layers (Figure 2F).
22. Centrifuge at 1750 *g* for 20 min at 4°C, using minimum acceleration and no braking.
23. After centrifugation, the liquid is divided into three layers, with pancreatic islets mainly in the middle layer, visible as a suspended pellet (Figure 2G). Carefully aspirate as much of the islet pellet as possible from the middle layer using a disposable pipette into a centrifuge tube containing 20 mL of Neutralization buffer.
24. Centrifuge the islets at 180 *g* for 1 min at 4°C (Figure 2H). Discard the supernatant, resuspend the pellet in 20 mL of Neutralization buffer, and vortex until the suspension is homogeneous.
25. Repeat step 24 three times to wash the pellet thoroughly and remove 1077.
26. Resuspend the pellet in 5 mL of RPMI 1640 medium containing 10% FBS and transfer to a 60 mm non-treated petri dish. Rinse the walls of the centrifuge tube with an additional 5 mL of medium and transfer all liquid to the petri dish.
27. Hand-pick islets and transfer them into the dish.
  a. Hand-pick islets using a bottom-illuminated stereomicroscope at 40X magnification.
  b. Use a 200 μL pipette gun to carefully aspirate appropriately sized islets, avoiding other tissues.
  c. Transfer each islet to a 35 mm dish containing 2 mL of islet medium.
  d. Gently blow through the dish several times to prevent islet clustering.

**CRITICAL:** Islets must be cultured in suspension, so ensure the 35 mm dish is non-treated or ultra-low attachment plate.

**Figure 3:**
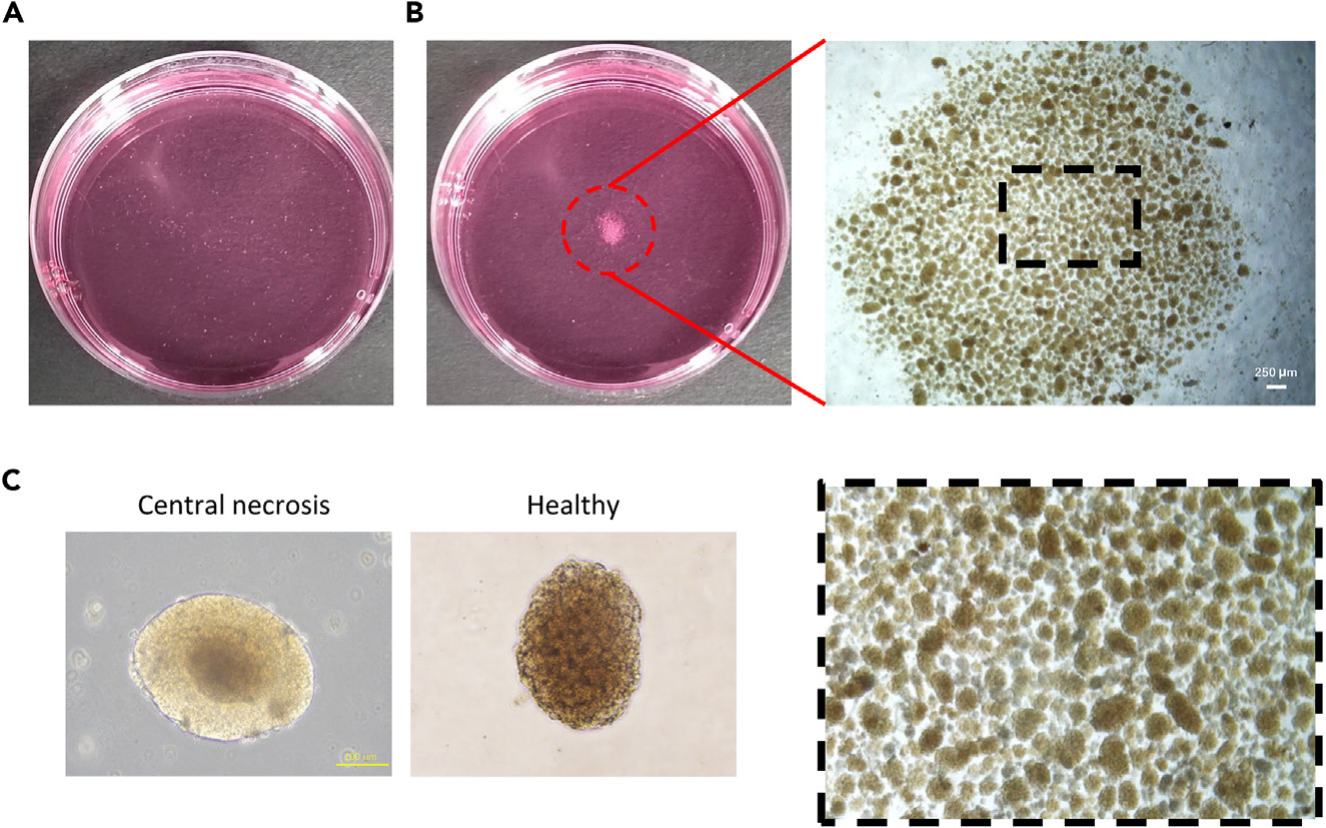
Representative images of purified islets (A) Nearly 100% pure islets obtained after manual handpicking. The surface of freshly isolated islets appears rough, likely attributed to disruption of the outer cell layer during enzymatic isolation procedures. (B) Shake the culture dish to gather the islets in the center of the dish. (C) Islets cultured for 12 h exhibit a smooth periphery, indicative of recovery from the stress from isolation. Occasionally, larger islets may display a dark central region.

28. Shake the dish and incubate it for subsequent experiments.
  a. After collecting all the islets, place the 35 mm dish on a level surface and gently shake it using a cross-shaking method to ensure even distribution of the islets (Figure 3A).
  b. Typically, a mature rat aged 8 weeks could yield 600–1000 islets following the procedures outlined in this protocol.
  c. To prevent the islets from aggregating in a 35 mm dish (Figure 3B), we generally place fewer than 500 islets in each well.
  d. Incubate the dish in a 37°C incubator with 5% CO_2_ for 14–16 h, suitable for subsequent experiments such as transplantation or staining.

**CRITICAL:** For transplantation purposes, select islets that are 50–225 μm in diameter, round or oval, and free of damage. Freshly isolated islets often have uneven edges, but 12 h incubation in the incubator smoothens their surfaces and improves their suitability for transplantation (Figure 3C). Aggregation of pancreatic islets in the medium can adversely affect their growth, so ensure they are distributed as evenly as possible (Figures 3A and 3B). During the manual selection of islets, take care to avoid aspirating acinar cells, as their presence in excess can compromise islet survival and growth.

***Note:*** When manually selecting acinar cells and islets, we can distinguish them by diameter and color. As for the diameter, most islets have a diameter of 25–200 μm, while the diameter of acinar cells is around 5–50 μm, with 95% of acinar cells being significantly smaller than islets. As for the color, under a bottom-illuminated stereomicroscope, magnified 20–40 times, islets appear yellow, with a dense structure that is completely opaque, whereas acinar cells are gray-black, with a loose structure that is somewhat transparent.

### Islets transplantation and IPGTT

**Timing: 4 h**

In this section, harvested islets are transplanted into the previously modeled diabetic mice via renal capsule delivery. After 21 days, IPGTTs are performed on the mice to confirm the function and efficacy of the islets.

**Figure 4:**
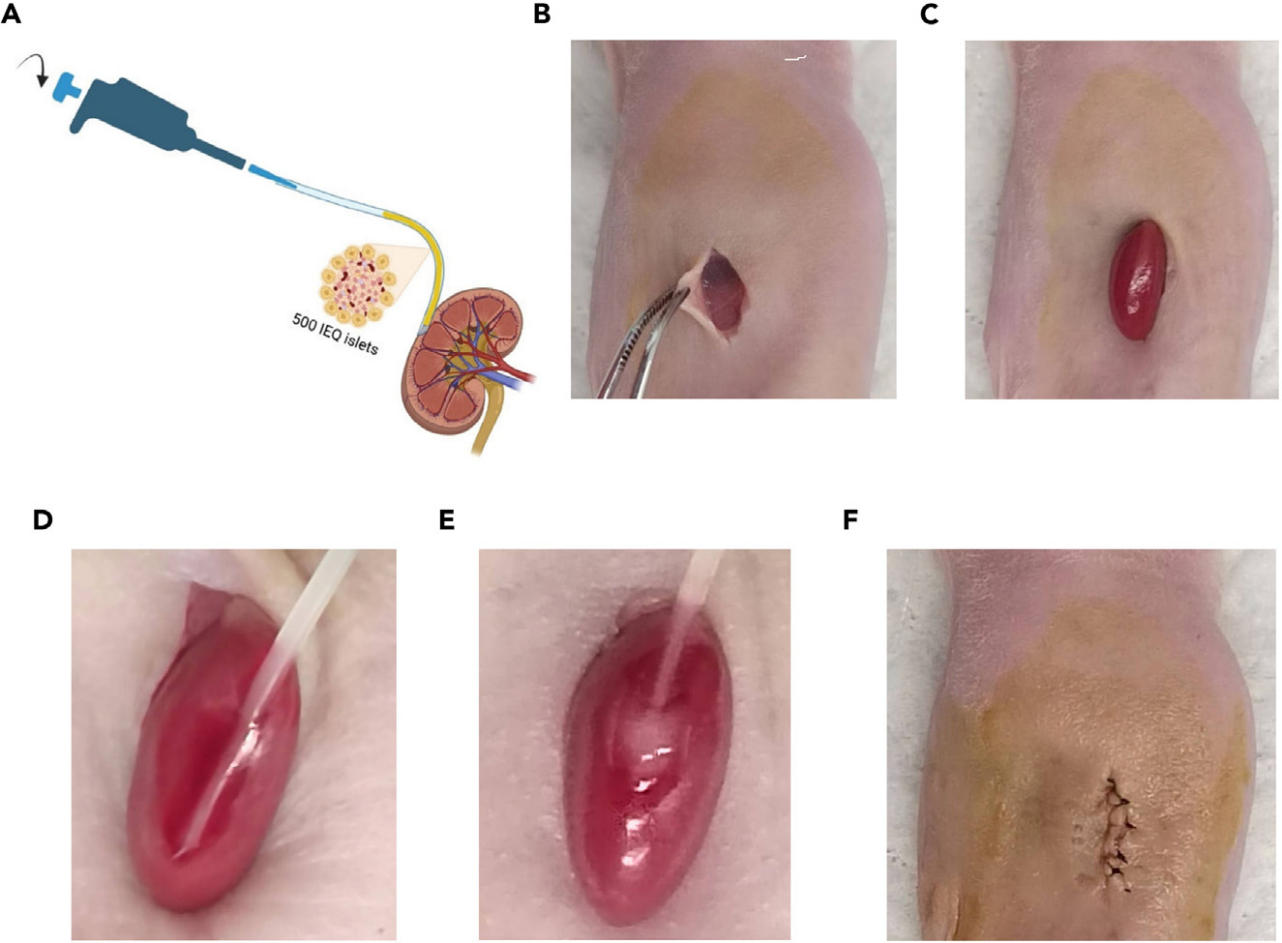
Surgical procedure for rat islets transplantation into nude mice (A) Preparation of a pipette and PE50 tube assembly for the transplantation of pancreatic islets. (B) An anesthetized mouse positioned pronely, with surgical precision scissors used to incise the skin around the kidney. (C) Gentle manipulation to extract the kidney through the incision. (D) Introduction of the sharp end of the PE50 tubing into the renal capsule. (E) Adjustment of the pipette volume to gradually deliver pancreatic islets from the PE50 tubing into the renal capsule. (F) Reinsertion of the kidney into the abdominal cavity followed by suturing of the incision site.

**Figure 5:**
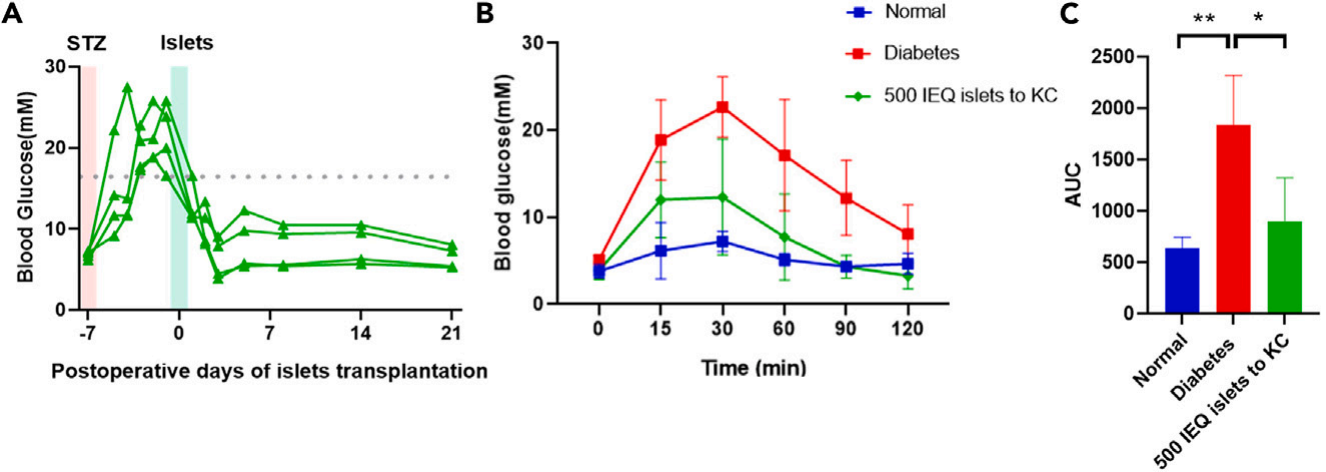
The function of rat islet transplanted into diabetes nude mice (A) Monitoring of blood glucose levels in nude mice over various days post-transplantation of rat islets. (B) IPGTT results of mice in different experimental groups. (C) IPGTT results of corresponding AUC values. All data are presented as mean ± SD. Statistical analysis included one-way ANOVA followed by Tukey’s test for multi-group comparisons. Two-tailed Student’s t-test was used for comparisons involving two groups. Significance levels are denoted as ∗, ∗∗, and ∗∗∗, indicating *p* values < 0.05, <0.01, and <0.001, respectively.

29. Sterilize surgical instruments (scissors, smooth forceps, and needle holder).
30. Prepare povidone-iodine, cotton swabs, suture needles, saline, and 1 mL syringes in advance.
31. Extract 500 IEQ of pancreatic islets into a PE50 tubing. One end of the PE50 tubing is connected to a 20 μL pipette tip and pipettor, and the other end is trimmed to a sharp point for insertion into the renal capsule, as depicted in Figure 4A.
32. Anesthetize diabetic mice with isoflurane in a prone position, securing the limbs. Roughly locate the left kidney position and disinfect the skin with povidone-iodine.
33. Deliver the islets into the renal capsule of diabetic mice.
  a. Make an incision above the left kidney (Figure 4B).
  b. Exteriorize the kidney (Figure 4C).
  c. Insert the sharp end of the PE50 tubing into the renal capsule (Figure 4D).
  d. Adjust the pipettor’s volume knob to slowly deliver pancreatic islets from the PE50 tubing into the renal capsule (Figure 4E).
  e. Once all islets are transferred, carefully withdraw the PE50 tubing from the renal capsule.
34. Carefully push the kidney back into the mouse’s abdomen, suture the surgical incision (muscle first, then skin), and disinfect the wound with povidone-iodine (Figure 4F). Terminate anesthesia and monitor the mouse until it regains consciousness.
35. Monitor mouse blood glucose levels once daily on days 1, 2, 3, 5, 8, 10, 14, and 21 post-transplantation (Figure 5A).
36. Conduct IPGTT on the mice.
  a. On the 21st day post-transplantation, fast the mice from the transplantation group, control group, and diabetic group for 12 h (with water provided).
  b. Intraperitoneally inject 2 mg/kg of D-glucose solution.
  c. Measure the blood glucose levels right before the intraperitoneal injection and at 15, 30, 60, 90, and 120 min thereafter.
  d. Area under the curve (AUC) calculations are performed for each group, and statistical analysis is conducted using ANOVA^2^ (Figures 5B and 5C).

## Expected outcomes

Factors such as age and weight significantly influence the yield of pancreatic islets, with our protocol typically yielding 800–1000 islets from the pancreas of one adult male SD rat. Evaluation of the islet quality involves assessing purity and morphology. Throughout experimental procedures such as digestion, centrifugation, and vortexing, islets inevitably sustain damage, characterized by uneven edges and damaged connective tissues at the periphery immediately after extraction.^3^ Consequently, freshly isolated islets are typically unsuitable for experiments requiring high-quality islets, such as transplantation.^4^ Islet quality improves after 12 h culture, during which the rough connective tissue on the surface sloughs off, and the thickness of the detached tissue is approximately 2–5 μm, resulting in smoother edges. However, extended culture beyond 24 h in the incubator carries the risk of aggregation and necrosis (Figure 3C), compromising islet viability and functionality for subsequent experiments.^5^

After 12 h culture of rat islets, 500 IEQ (islet equivalents) were transplanted into the renal subcapsular space. Blood glucose levels were monitored at various time points post-transplantation. On the first day post-transplantation, 75% of nude mice exhibited glucose levels restored to normal (<16.7 mM), and by the second day, all nude mice showed glucose levels reduced to <16.7 mM (Figure 5A). On the 21st day post-transplantation, an intraperitoneal glucose tolerance test (IPGTT) was conducted, revealing a significantly lower AUC in the transplantation group compared to the diabetic group^6^ (Figures 5B and 5C).

## Limitations

Numerous steps in the experimental process can significantly influence the yield and quality of pancreatic islets, including collagenase selection, digestion duration, 1077 gradient dwell time, centrifugation, vortexing, and shaking.^5^ While efforts have been made to optimize these operations to minimize islet loss and damage, complete avoidance has not been achieved. Consequently, the manual selection of intact and non-necrotic islets remains essential, despite being time-consuming and labor-intensive, there is no alternative solution with higher efficiency available.^7^

Furthermore, variations in centrifuge rotor radii lead to significant differences in centrifugal force, necessitating the use of relative centrifugal force (rcf) to standardize centrifugation outcomes, rather than relying solely on rotational speed (rpm). Therefore, this protocol specifies centrifugation in terms of gravitational force (g) across all units to ensure consistency in centrifugal effects.^3^

## Troubleshooting

### Problem 1

Difficulty with bile duct intubation.

### Potential solution

A critical requirement for effective pancreatic perfusion is proper intubation of the common bile duct. While the rat’s common bile duct is thicker compared to mice, there remains a risk of inadvertently inserting the needle around rather than into the duct, leading to localized perfusion of the collagenase P working solution. Multiple attempts at intubation can also result in duct damage and failure to adequately perfuse the pancreas. It is advisable to first remove surrounding adipose tissue to facilitate clear identification of the bile duct. Using ophthalmic curved forceps to gently grasp the duodenum near the upper Oddi sphincter can further aid in exposing the bile duct.

Alternatively, an intravenous needle can be modified by breaking it at a 90° angle with the beveled side facing upward. This needle can then be inserted at an approximate 20° angle to the bile duct, allowing for gentle injection of approximately 100 μL of Collagenase P working fluid to fill the duct. Subsequently, the needle should be advanced parallel to the bile duct for approximately 1.5 cm to ensure proper placement and avoid damage (Steps 12 and 13).

### Problem 2

Inadequate perfusion of the pancreas.

### Potential solution

It is crucial to identify the leakage point of collagenase working fluid when pancreatic perfusion is still insufficient even after the bile duct has been correctly intubated. If bowel expansion is observed during perfusion, indicating that the hemostatic clip is not tightly clamped or is improperly positioned in the duodenum, the junction of Vater’s papilla at the pancreatic duct and duodenum (illustrated in Figure 1D) should be inspected and adjusted to ensure proper clamp placement.

Given the thicker bile duct in rats, the depth of needle insertion should not be too shallow (greater than 1.5 cm). It is essential to use hemostatic forceps to secure the bile duct and the inserted needle to prevent fluid reflux and subsequent leakage during perfusion. Moreover, the rate of collagenase injection should be carefully controlled; injecting too quickly may increase local pancreatic duct pressure, potentially causing duct rupture, while injecting too slowly may damage pancreatic epithelium, both scenarios leading to collagenase working fluid leakage (Step 9).

### Problem 3

Low pancreatic islet production.

### Potential solution

Digestion time plays a pivotal role in determining islet yield, influenced by variables such as rat strain, age, and weight. Experimenters are advised to conduct preliminary tests to determine the optimal digestion time tailored to their specific experimental conditions, following this protocol. During islet purification, minimizing cell adhesion to equipment like centrifuge tubes, pipette tips, filters, and pipettes is crucial. After gradient centrifugation, approximately 10 mL needs to be aspirated from the middle layer of the liquid. Increasing this volume can increase pancreatic islet production, although an additional 1077 is also aspirated at this time. To reduce the damage of 1077 to pancreatic islets, at least three subsequent washes are crucial for effectively removing residual 1077 (Steps 23 to 25).

### Problem 4

Insufficient or excessive digestion of the pancreas.

### Potential solution

In the manual selection of pancreatic islets under the microscope, the degree of digestion can be gauged by observing the size of the islets. The majority of large pancreatic islets indicate insufficient digestion and require an extension of digestion time. On the contrary, the majority of small islets indicate excessive digestion and should shorten digestion time. This qualitative assessment is crucial for optimizing the digestion process to obtain pancreatic islets of appropriate size and quality for experimental purposes (Step 23).

In this protocol, we use 8-week-old rats to isolate islets, with an optimal digestion time of 30 min. When the digestion time is 20 min, each rat yields fewer than 50 islets, and most islets are not fully separated. When the digestion time is 25 min, each rat yields 200–400 islets, and the islet diameter is relatively large. When the digestion time is 28–32 min, the islet yield is maximized, with an average of 600–1000 islets per rat, and the islet diameters are evenly distributed between 25–200 μm. When the digestion time is 40 min, the islet yield decreases, and the overall islet diameter is smaller, showing signs of over-digestion.

## Resource availability

### Lead contact

Further information and requests for resources and reagents should be directed to and will be fulfilled by the lead contact, Kai Wang (kai.wang88@pku.edu.cn).

### Technical contact

Technical questions about this protocol should be directed to the technical contact, Dayan Li (2311110036@stu.pku.edu.cn).

### Materials availability

This study did not generate new unique reagents.

### Data and code availability

This study did not generate any unique datasets or code.

## Acknowledgments

This work was funded by the National Key R&D Program of China (2022YFA1104800); the Beijing Natural Science Foundation (JQ23029 and L234024); the Beijing Nova Program (20220484100 and 20230484448); the National Natural Science Foundation of China (82370514); the Beijing Municipal Science & Technology Commission (Z231100007223001); the open research fund of State Key Laboratory of Cardiovascular Disease, Fuwai Hospital (2022KF-04); the Clinical Medicine Plus X-Young Scholars Project, Peking University (PKU2022LCXQ003); the Emerging Engineering Interdisciplinary-Young Scholars Project, Peking University; the Fundamental Research Funds for the Central Universities (PKU2023XGK011); the open research fund of State Key Laboratory of Digital Medical Engineering, Southeast University (2023K-01); and the open research fund of Beijing Key Laboratory of Metabolic Disorder Related Cardiovascular Disease, Beijing, PR China (DXWL2023-01).

## Author contributions

Methodology, D.L. and W.K.; experiments, D.L., W.Z., B.S., and W.K.; writing, D.L., Y.H., W.K., and X.W.; funding acquisition, K.W.; supervision, X.W. and K.W.

## Declaration of interests

The authors declare no competing interests.
